# A Patient-Centered Evaluation of Meaningful Change on the 32-Item Motor Function Measure in Spinal Muscular Atrophy Using Qualitative and Quantitative Data

**DOI:** 10.3389/fneur.2021.770423

**Published:** 2022-01-17

**Authors:** Tina Duong, Hannah Staunton, Jessica Braid, Aurelie Barriere, Ben Trzaskoma, Ling Gao, Tom Willgoss, Rosangel Cruz, Nicole Gusset, Ksenija Gorni, Sharan Randhawa, Lida Yang, Carole Vuillerot

**Affiliations:** ^1^Department of Neurology, Stanford University, Stanford, CA, United States; ^2^Roche Products Limited, Welwyn Garden City, United Kingdom; ^3^Department of Pediatric Physical Medicine and Rehabilitation, Hôpital Mère Enfant, Centre Hospitalier Universitaire (CHU)-Lyon, Lyon University, Lyon, France; ^4^Genentech Inc., South San Francisco, CA, United States; ^5^Analystat Corporation, Point Roberts, WA, United States; ^6^Cure SMA, Elk Grove Village, IL, United States; ^7^SMA Europe, Freiburg, Germany; ^8^SMA Schweiz, Swiss Patient Organisation for Spinal Muscular Atrophy, Heimberg, Switzerland; ^9^Product Development Medical Affairs, Neuroscience and Rare Disease, F. Hoffmann-La Roche Ltd., Basel, Switzerland; ^10^Adelphi Values, Patient-Centered Outcomes, Adelphi Mill, Bollington, United Kingdom; ^11^Charles River Associates Inc., Zurich, Switzerland; ^12^Neuromyogen Institute, CNRS UMR 5310 INSERM U1217, Université de Lyon, Lyon, France

**Keywords:** 32-item Motor Function Measure (MFM32), spinal muscular atrophy, meaningful change, qualitative interviews, online survey, anchor-based methods

## Abstract

The 32-item Motor Function Measure (MFM32) is an assessment of motor function used to evaluate fine and gross motor ability in patients with neuromuscular disorders, including spinal muscular atrophy (SMA). Reliability and validity of the MFM32 have been documented in individuals with SMA. Through semi-structured qualitative interviews (*N* = 40) and an online survey in eight countries (*N* = 217) with individuals with Types 2 and 3 SMA aged 2–59 years old and caregivers, the meaning of changes on a patient-friendly version of the MFM32 was explored. In an independent analysis of clinical trial data, anchor- and distribution-based analyses were conducted in a sample of individuals with Type 2 and non-ambulant Type 3 SMA to estimate patient-centered quantitative MFM32 meaningful change thresholds. The results from this study demonstrate that, based on patient and caregiver insights, maintaining functional ability as assessed by a patient-friendly version of the MFM32 is an important outcome. Quantitative analyses using multiple anchors (median age range of 5–8 years old across anchor groups) indicated that an ~3-point improvement in MFM32 total score represents meaningful change at the individual patient level. Overall, the qualitative and quantitative findings from this study support the importance of examining a range of meaningful change thresholds on the MFM32 including ≥0 points change reflecting stabilization or improvement and ≥3 points change reflecting a higher threshold of improvement. Future research is needed to explore quantitative differences in meaningful change on the MFM32 based on age and functional subgroups.

## Introduction

Spinal muscular atrophy (SMA) is a rare, autosomal, recessive neuromuscular disease characterized by slow, progressive muscle weakness and atrophy of the skeletal muscles. The phenotypic spectrum is classically divided into four subtypes (1–4 [most severe to least severe]) based on age of onset and the maximum motor milestone achieved (1). However, this classification is evolving to focus on milestone achievement (non-sitters, sitters, walkers) (2). When focusing on individuals with Types 2 and 3 SMA, symptom presentation in individuals with Type 2 SMA occurs between ages 7–18 months and after 18 months of age for individuals with Type 3 SMA (1). Types 2 and 3 SMA are more heterogeneous and less severe than Type 1 SMA, in which untreated babies are unable to sit (3). Some individuals with Type 3 SMA are able to stand and walk independently, although these abilities may be lost as the disease progresses (3, 4). Individuals with Types 2 and 3 SMA commonly also have scoliosis and contractures that impact motor abilities (5, 6).

Due to the different clinical presentations of SMA symptoms across the population, numerous clinical outcome assessments (COAs) have been developed to capture the full range of symptom presentations and primarily focus on assessing change in motor function (7). The 32-item Motor Function Measure (MFM32) is a clinician-reported outcome (ClinRO) used to evaluate fine and gross motor ability in individuals with neuromuscular disorders, including SMA (8). The MFM32 was developed by clinical experts to assess important motor constructs across a range of functional abilities through items associated with standing and transfers (e.g., walking and standing up from sitting), proximal and axial function (e.g., rolling, sitting) and distal motor function (e.g., finger dexterity, hand function).

The MFM32 is validated for use in individuals with neuromuscular disorders, including those with Types 2 and 3 SMA (8, 9) and has been found to be better targeted to weaker patients with a more progressed disease (7). Although the MFM32 has demonstrated acceptable reliability, validity and responsiveness (10), there remains an important need to further understand meaningful change on the scale from a patient-centered perspective using both qualitative and quantitative methods.

The US Food and Drug Administration Patient Focused Drug Development (PFDD) Guidance 3 Discussion Document (11) emphasizes the importance of establishing meaningful change on COAs at the individual patient level, rather than focusing on the clinical meaningfulness of between-group-level differences which do not provide insights into the level of change an individual has experienced. Institute for Quality and Efficiency in Health Care (IQWiG) General Methods guidance in Germany (12) also refer to the importance of assessing clinical meaningfulness at the individual patient-level. Anchor-based estimates that use a patient-centered external criterion to assess the level of meaningful change an individual has experienced are preferred, with distribution-based estimates providing supportive evidence of change beyond a degree of measurement error. Approaches that complement these quantitative analyses, such as qualitative insights from patients and caregivers, are increasingly endorsed in order to provide context to what a change on a ClinRO means for the target population (13, 14).

**Figure 1:**
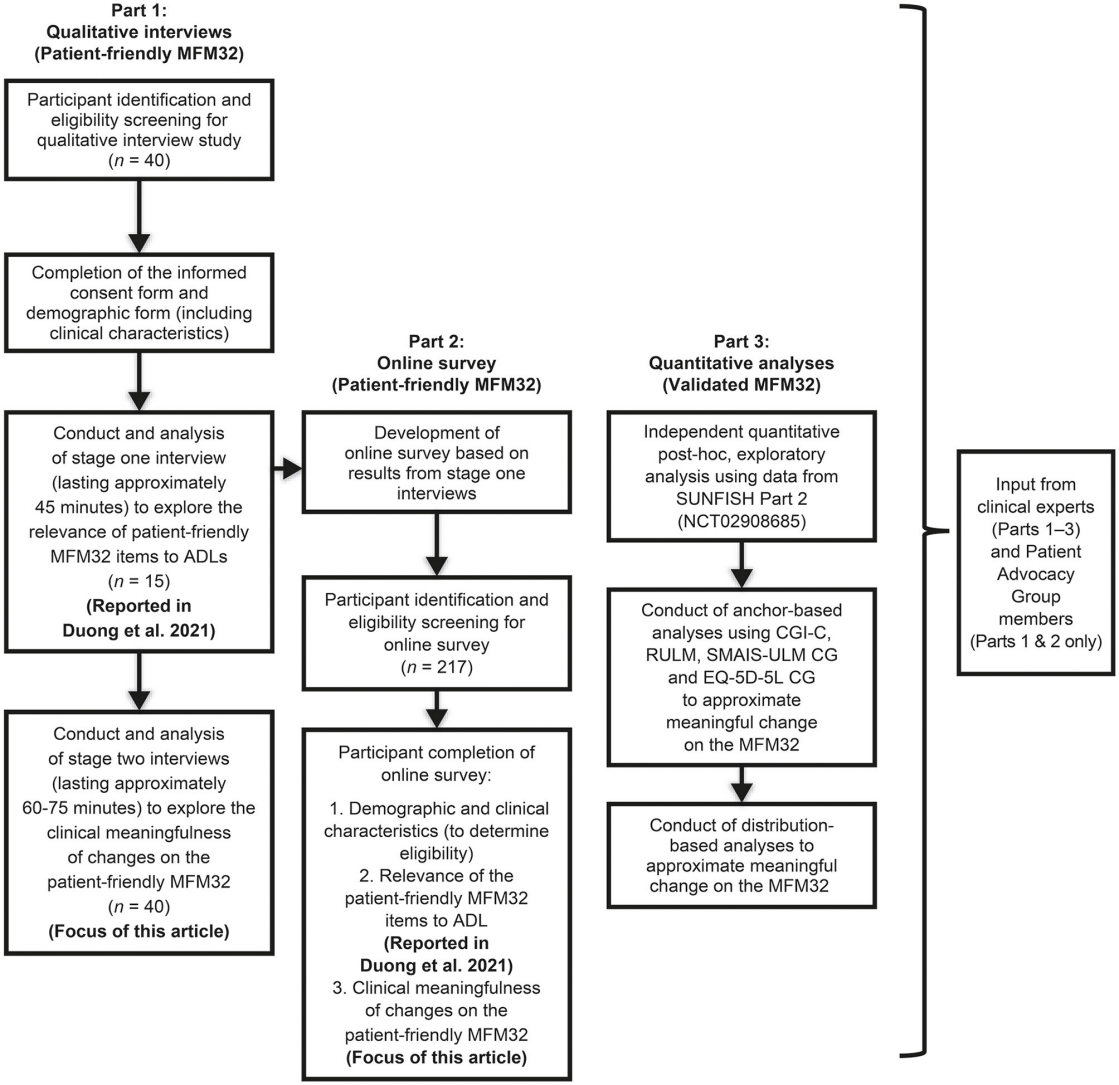
Study design. MFM32, 32-item Motor Function Measure; ADLs, activities of daily living; CGI-C, Clinical Global Impression of Change; RULM, Revised Upper Limb Module; SMAIS-ULM CG, SMA Independence Scale Upper Limb Module Caregiver report; EQ-5D-5L CG, EuroQoL 5D-5L Caregiver report. This figure was adapted from Duong et al. (15).

In this work, we describe a comprehensive effort to provide a patient-centered evaluation of meaningful change at the individual patient-level on the MFM32 through both qualitative and quantitative methodologies (Figure 1). The first component consisted of qualitative semi-structured interviews (Part 1), which were supplemented by an online survey (Part 2). In an independent quantitative analysis (Part 3), we utilized data from SUNFISH Part 2 (NCT02908685), a randomized, double-blind, placebo-controlled clinical trial, to conduct exploratory *post-hoc* anchor- and distribution-based analyses to estimate thresholds for meaningful change from a patient-centered perspective.

## Methods

In the validated version of the MFM32, the scoring of each item uses a 4-point Likert scale (0–3), with higher scores associated with better motor function abilities (8). The 32 scores are summed and then transformed onto a 0–100 scale to yield a total score expressed as the percentage of the maximum possible score; the lower the total score, the more severe the impairment. For the purpose of the qualitative interviews and online survey, a self-reported, patient-friendly version of the MFM32 items, developed in collaboration with members of the MFM group and patient advocacy groups, was completed by patients/caregivers to provide an approximate understanding of the individual's current level of motor function ability. The patient-friendly version of the MFM32 was created to reduce clinical terminology whilst ensuring that the emphasis on the specific ability being assessed was maintained. This was done to ensure patient understanding of the concepts assessed. An overview of the patient-friendly MFM32 measure used in the interviews (maintaining 0–3 point scale) and the survey (reducing to can/cannot do responses) has been previously described (15). Supplementary Table 1 includes a comparison of the patient-friendly MFM32 items used in the qualitative interviews and online survey and the MFM32 clinical items used in the quantitative analysis.

### Part 1. Qualitative Interviews Using a Patient-Friendly Version of the MFM32 Assessment

In-depth, semi-structured qualitative telephone/WebEx interviews were conducted with individuals with Type 2 SMA and ambulant (i.e., able to walk unassisted for 10 meters or more) and non-ambulant (i.e., unable to walk unassisted for 10 meters or more) Type 3 SMA and their caregivers (ambulatory status was self-assessed). In brief, eligible individuals with SMA were required to be aged 12–60 years old and caregivers had to be 18 years old or above and care for an individual aged 2–60 years old. Additional eligibility criteria are described in the Supplementary Methods section. Telephone interviews lasting between 60 [*n* = 15 who took part in an activities of daily living (ADL) interview described in (15)] to 75 min (*n* = 25 new participants) were conducted with individuals with SMA or caregivers of individuals with SMA from the USA. During the interview, participants were asked questions at an item and total score level. At the item level, in order to focus the discussion, the last three items where the participant scored a 2 (able to perform the ability/movement but with some help, slowly, without complete control or can't hold it for long), 1 (start the ability/movement but unable to finish it), and 0 (unable to start an ability or movement) were identified. The participants were then asked to discuss what it would mean to improve from their current level of functioning, and what impact this change might have on their ability to perform ADL. Participants were also asked to reflect on improving, remaining stable (i.e., maintaining current level of function) and declining on the patient-friendly MFM32 total score over a 1-year period.

The meaningful change interviews were conducted between February 2020 and May 2020. Interviews were audio recorded and transcribed *verbatim*. Data were subjected to thematic analysis, a process by which researchers review interview transcripts to identify, analyze and interpret patterns (or “themes”) within qualitative data by assigning “codes,” which was facilitated using ATLAS.Ti software (16). The study was approved by Copernicus Group IRB (20192527) and written informed consent was obtained from participants aged ≥18 years old and written informed consent was provided by caregivers for individuals aged 12–17 years old prior to the interviews, as well as obtaining assent from adolescents. Participants were reimbursed for their time in line with US fair market value for interview research. Interviews were conducted by experienced qualitative researchers.

### Part 2. Online Survey Using a Patient-Friendly Version of the MFM32 Assessment

The online survey was conducted following feedback from the patient advocacy groups involved in this project regarding the importance of the inclusion of additional countries outside the US. The same eligibility criteria used in the qualitative interview study were applied for the online survey. Participants completed questions from the online survey relating to the MFM32 items relationship to ADL (15) and secondly relating to meaningful change. Questions included the importance of maintaining a similar level of functioning over a 1-year period, improving on the abilities currently able to perform (i.e., responded “can do”) and improving on a selection of abilities currently unable to perform (i.e., first three items where participant responded “cannot do”) based on the patient-friendly MFM32.

The online survey was completed by participants between January to April 2020. It was conducted following market research principles and hence did not receive ethical approval but was consistent with British Healthcare Business Intelligence Association Adverse Event Reporting in Market Research as well as General Data Protection Regulation guidelines and guidelines set by ESOMAR and EphrMRA for all European research. In addition, data checks (e.g., avoidance of duplicate participants) were in place to ensure data integrity. A tick box indicating consent to participate was included in the survey prior to completion. Assent was provided by caregivers prior to survey completion for individuals aged <18 years old. Upon completion of the survey, participants were reimbursed for their time in line with geographical fair market value rates for survey research which was not linked to the patient or caregiver survey responses.

### Part 3. Quantitative Analyses Using the Validated MFM32 Assessment

Analyses were performed using MFM32 data from Part 2 (up to 52 weeks) of the SUNFISH clinical trial (*N* = 180 target sample). The SUNFISH study is a multicenter, randomized, double-blind, placebo-controlled, Phase 2/3 study to assess the safety, tolerability, pharmacokinetics, pharmacodynamics, and efficacy of risdiplam in a broad adult and pediatric population aged 2–25 years old with Type 2 and non-ambulant Type 3 SMA, including patients with contractures and scoliosis. Refer to Mercuri et al. (17) for methods and results of the SUNFISH Part 2 clinical trial. For this research, the risdiplam and placebo 52-week data from SUNFISH Part 2 were pooled as the aim of this analysis was to define a meaningful score change on the MFM32 for future research rather than to evaluate treatment efficacy (18, 19). The analyses were conducted as *post-hoc* exploratory analyses. Thresholds for meaningful individual patient-level change were estimated based on methods recommended in the FDA Patient-Reported Outcomes guidance (2009) (20) and PFDD Guidance 3 Discussion Document (2018) (11). In line with this guidance, anchor-based estimates were the primary approach, while distribution-based estimates were considered supportive.

#### Anchor-Based Methods

Anchor-based methods use an external criterion of known relevance to define individuals who have experienced a meaningful change in their condition (11). In line with industry guidance, a variety of anchors were evaluated based on their relevance to patients and suitability as an anchor according to their correlation with the MFM32 (21). Typically correlations between the anchor and target measure should ideally exceed 0.30 to support interpretation (21). However, it is acknowledged that the clinical relevance of the anchor is also a key consideration and >0.50 has been deemed a very high correlation, >0.40 a high correlation, >0.21 a moderate correlation and ≤0.20 a weak correlation (22). Further evaluation of anchor suitability was explored through empirical cumulative distribution function (CDF) plots (11) to ensure that the anchor groups adequately discriminated between individuals improving and worsening on the MFM32. Two clinical anchors assessing overall health status and motor function [the Clinical Global Impression of Change (CGI-C) scale and the Revised Upper Limb Module (RULM)] and two caregiver anchors assessing everyday activities [SMA Independence Scale Upper Limb Module (SMAIS-ULM) caregiver report and EuroQol 5D-5L (EQ-5D-5L) caregiver report, a self-care item assessing the caregiver's perspective of the individual's ability to wash and dress] were identified for the analysis. The anchors are further described in Table 1.

**Table 1:** Spearman's rank correlation coefficient correlations between the MFM32 and target anchor.

**Anchor**	**Description of scale and clinical relevance**	**Correlation at baseline with the MFM32**	**Correlation at Week 52 with the MFM32**	**Correlation of change from baseline to Week 52 with the MFM32**
CGI-C	Single item assessing change in the patient's overall health from baseline, rated by clinicians at Week 52. Response options range from very much improved (1) to very much worse (7).	n/a	n/a	−0.48 (*n* = 159)
RULM	The RULM assesses the motor performance of the upper limbs in SMA. It consists of 19 scoreable items that test proximal and distal motor functions of the arm in patients with SMA (23). Higher scores indicate better motor function ability.	0.85 (*n* = 171)	0.87 (*n* = 162)	0.50 (*n* = 162)
SMAIS-ULM CG	The SMAIS-ULM was developed specifically for SMA in order to assess function-related independence (24). The SMAIS-ULM total score consists of 22 items focused on upper-limb-related ADLs, with higher scores indicating greater independence.	0.69 (*n* = 171)	0.70 (*n* = 163)	0.22 (*n* = 161)
EQ-5D-5L CG self-care item	The EQ-5D-5L is a generic self- or caregiver-reported health status questionnaire that is used to calculate a health utility score for use in health economic analysis (25). The self-care item is scored from 1 (I have no problems washing or dressing myself) to 5 (I am unable to wash or dress myself) scale.	−0.51 (*n* = 168)	−0.64 (*n* = 167)	−0.20 (*n* = 162)

*MFM32, 32-item Motor Function Measure; CGI-C, Clinical Global Impression of Change Scale; RULM, Revised Upper Limb Module; SMA, spinal muscular atrophy; SMAIS-ULM CG, SMA Independence Scale Upper Limb Module Caregiver Report; ADLs, activities of daily living; EQ-5D-5L CG, EuroQol 5D-5L Caregiver Report*.

In line with Copay et al. (18) and Howells et al. (26), individual patient improvement meaningful change estimates (also called within-patient estimates) were determined. The least squares (LS) mean change in the MFM32 total score was derived from a repeated measures model by identifying patients who had experienced the following level of change from baseline to Week 52 on each of the target anchors individually:

- Improvement (i.e., combined minimally, much and very much improved groups) on the CGI-C.
- Improvement on the RULM defined as ≥2 and ≥3 points in RULM total score (27, 28).
- Improvement on the SMAIS-ULM reported by caregivers defined as ≥3 points in 22-item upper limb total score (24).
- Improvement on the EQ-5D-5L self-care item reported by caregivers defined as improved by ≥1 point.

LS mean change scores of the MFM32 over 52 weeks corresponding to the pre-specified anchor levels were estimated using the change in MFM32 as the dependent variable and the change in anchor measure (i.e., CGI-C, RULM, SMAIS-ULM caregiver report and EQ-5D-5L caregiver report self-care item) as a categorical predictor, with age category (2–5, 6–11, and 12–25 years old), SMA type (Type 2 and non-ambulant Type 3), years of disease duration, study visit, study treatment arm and baseline MFM32 as covariates. The LS means were extracted for the improved level of the anchor variable from a repeated measures model.

#### Distribution-Based Methods

Distribution-based methods assess the distribution of scores on the target measure at a single time point to classify the size of meaningful change considered to have occurred beyond a degree of measurement error, rather than the statistical or clinical significance of that change. In addition to the anchor-based methods described, ± 1 standard error of measurement (SEM) on the MFM32 ($SEM=SD_{BL}*\sqrt{1-reliability}$) at baseline using Cronbach's alpha (29) to estimate reliability was conducted. The value of 1 SEM has previously been found to correspond to anchor-based meaningful change results (30). Furthermore, 0.5 standard deviation (SD) and 0.2 SD on the MFM32 at baseline were evaluated to approximate a moderate and small effect size, respectively (18).

#### Triangulation

Final meaningful change estimates were selected using triangulation (i.e., integrating qualitative and quantitative insights in order to propose a range of estimates) (31). The triangulation approach involves researchers collectively evaluating all the estimates generated across the different methods and selecting the respective range of values for the MFM32 where there is convergence. The most consideration was given to estimates generated from qualitative studies and anchor-based analyses as these are tied to the patient/clinical perspective, with distribution-based estimates considered as exploratory.

## Results

### Part 1. Qualitative Interview Results Using a Patient-Friendly Version of the MFM32 Assessment

Twenty-eight individuals with SMA and 12 caregivers were interviewed in total (see Table 2) and no individual/caregiver dyads were recruited (i.e., each response pertained to unique individuals). The mean age of individuals (including individuals with SMA who were reported on by caregivers) was 19.7 years (range 3–45 years) and 68% were female. Forty-eight percent of individuals had Type 2 SMA, while the remaining population included both individuals with ambulant Type 3 (30%) and non-ambulant Type 3 (23%) SMA. The majority (90%) of individuals were taking nusinersen (SPINRAZA^®^) treatment at the time of interview, all of whom were in the maintenance dosing phase of nusinersen treatment.

**Table 2:** Interview and online survey sample demographic characteristics.

**Individuals with SMA—interview sample demographic characteristics reported by the individual (*n* = 28) and caregiver (*n* = 12)**	**Total sample (*N* = 40)**
**Age**, mean years (min–max)	19.7 (3–45)
**Gender**, *n* (%)	
Male	13 (32.5)
Female	27 (67.5)
**SMA type**, *n* (%)	
Type 2	19 (47.5)
Type 3, non-ambulant	9 (22.5)
Type 3, ambulant	12 (30.0)
**Currently receiving/taking treatment to manage SMA**, *n* (%)	
No	4 (10.0)
Yes	36 (90.0)
**Self-reported changes to severity of SMA over the last year**, *n* (%)	
Improved	12 (30.0)
Stable/unchanged	23 (57.5)
Worse	5 (12.5)
**Individuals with SMA—online survey sample demographic characteristics reported by the individual (*****n*** **=119) and caregiver (*****n*** **=** **98)**	**Total sample (*****N*** **=** **217)**
**Country**, *n* (individuals, caregivers)	217 (119, 98)
USA	30 (17, 13)
Canada	23 (17, 6)
France	28 (15, 13)
UK	22 (8, 14)
Germany	20 (13, 7)
Italy	31 (16, 15)
Spain	32 (17, 15)
Poland	31 (16, 15)
**Age**, mean years (min–max)	27 (2–59)
**Gender**, *n* (%)	
Male	83 (38.2)
Female	134 (61.8)
**SMA type**, *n* (%)	
Type 2	116 (53.5)
Type 3, non-ambulant	51 (23.5)
Type 3, ambulant	50 (23.0)
**Currently receiving/taking treatment to manage SMA**, *n* (%)	
Discontinued	11 (5.1)
No	104 (47.9)
Yes	102 (47.0)
**Self-reported changes to severity of SMA over the last year**, *n* (%)	
Improved	54 (24.9)
Stable/unchanged	74 (34.1)
Worse	89 (41.0)

*SMA, spinal muscular atrophy. USA, United States of America; UK, United Kingdom. This table was adapted from Duong et al. (15)*.

The mean age of caregivers was 42.5 years (range 31–57 years) and 100% were female. The majority (92%) of caregivers provided care for one individual with SMA and all caregivers were the parent or legal guardian of the individual. On average caregivers self-reported providing 86.2 h (range 5–168 h) per week of care, though this varied depending on SMA type, with caregivers of individuals with Type 2 on average spending more time providing care when compared with caregivers of individuals with Type 3 SMA.

The importance of a single-point improvement at the item level on the patient-friendly version of the MFM32 was discussed 287 times across the 40 interviews. The improvement was considered important to the individual with SMA in most instances (244 out of the 287 times this was discussed, 85%), regardless of whether the participant scored 0, 1 or 2 on the item. At the item level 70% of participants (*n* = 28/40) indicated that small improvements on multiple items would be preferable, because of the potential incremental gains across different abilities. Twenty percent of participants (*n* = 8/40) indicated a preference for larger improvements on a single item, due to the fact the change may be more noticeable. The remaining 10% of participants (*n* = 4/40) did not have a preference for smaller vs. larger changes. In terms of deterioration, the sample was divided with regards to a preference for a smaller deterioration on several items (53%, *n* = 21/40) vs. a larger deterioration on a single item (45%, *n* = 18/40). The remaining participant's preference (3%, *n* = 1/40) was unclear.

**Figure 2:**
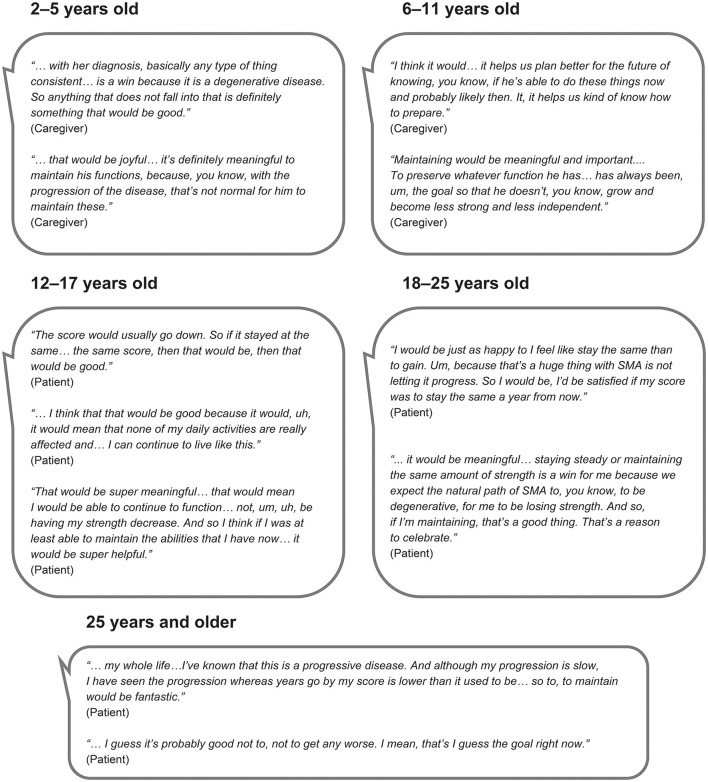
Quotes from the qualitative interviews illustrating the importance of maintaining functional ability over a 1-year period as measured by the patient-friendly MFM32 by caregivers and individuals with Type 2 and non-ambulant and ambulant Type 3 SMA.

The importance of maintenance, improvement and deterioration on the patient-friendly MFM32 total score, over a 1-year period, was discussed with all participants (100%, *n* = 40/40). As expected, the vast majority of participants considered improvement (100%, *n* = 40/40) and deterioration (98%, *n* = 39/40) on the patient-friendly MFM32 to be important. Similarly, the majority of participants (98%, *n* = 39/40) stated that maintaining their/the individual's motor function ability over a 1-year period as measured by the patient-friendly MFM32, would be a meaningful outcome (Figure 2). While maintaining motor function ability on the patient-friendly MFM32 was deemed to be important across the participants who participated in the interviews, there were important age-specific insights (Figure 2). For caregivers of infants and children, many descriptions focused on the future and the inevitable progression of the disease thus meaning that preserving function as early as possible was considered meaningful. Adolescents and adults described the importance of maintaining current ability from the perspective of having already lost function and highlighted their awareness of the future continued decline in performance on functional scales, leading to stabilization being a meaningful goal.

### Part 2. Online Survey Results Using a Patient-Friendly Version of the MFM32 Assessment

One hundred and nineteen individuals with SMA and 98 caregivers completed the online survey. Participants were evenly distributed geographically, with 20–32 responses per country, and no individual/caregiver dyads were recruited (i.e., each response pertained to unique individuals).

In the total sample, including individuals with SMA and caregivers reporting on individuals with SMA, the mean age of individuals with SMA was 27 years (range 2–59 years) and 62% were female (see Table 2). Over half of the individuals (*n* = 116) had Type 2 SMA. Almost half of the participants (*n* = 104) were treatment naïve, with the remaining participants currently in the maintenance phase of nusinersen treatment or having discontinued. Of the 98 caregivers surveyed, 73% provided care for one individual with SMA. Caregivers self-reported spending an average of 78.6 h (range 0–168 h) per week providing assistance to an individual/individuals with SMA. Caregivers of individuals with Type 2 SMA reported spending more time providing care on average when compared with caregivers of individuals with Type 3 SMA.

Confirmatory findings to the interviews were obtained in the online survey. A total of 196 participants (90%, *n* = 196/217) selected “yes” that maintaining a similar level of functional ability based on the patient-friendly MFM32 over a 1-year period would be a meaningful outcome. When asked about the importance of this outcome in daily life, participants selected a variety of pre-specified options, including “maintaining current quality of life” (92%, *n* = 181/196), “feeling reassured that the disease was not progressing” (87%, *n* = 170/196), the “ability to maintain current levels of independence” (85%, *n* = 166/196), the “ability to continue to perform certain tasks for the same length of time” (81%, *n* = 159/196) and “other” (10%, *n* = 19/196). The two most frequently reported “other” responses included “improving/maintaining mental health/less fear or concern” (53%, *n* = 10/19) and “demonstrating slowing of disease progression” (16%, *n* = 3/19). Of the remaining participants, 7% (*n* = 14/217) indicated that they were not sure whether maintaining their/the individual's functional ability would be a meaningful outcome and 3% (*n* = 7/217) indicated that they did not agree that maintaining their/the individual's functioning was a meaningful outcome. The most frequently reported reason for this disagreement provided by participants related to the expectation of treatments to offer improvement in functional ability (57%, *n* = 4/7).

In addition, 98% of participants (*n* = 213/217) selected “yes” when asked if some level of improvement in their ability to complete patient-friendly MFM32 items they are currently able to do would be a meaningful outcome, with the remaining 2% unsure (*n* = 4/217). When probed on the importance of experiencing some level of improvement via pre-specified response options, participants indicated that this would “help maintain their level of independence” (90%, *n* = 192/213) and their “current quality of life” (89%, *n* = 189/213), with 26% selecting “other reasons”. The two most frequently reported “other” responses included “improve independence/relieve caregivers” (*n* =23) and “more hope/boost of confidence meaning less anxiety about the future” (*n* = 15). When asked about some level of improvement for the first three items where the participant selected “cannot do,” 94% (*n* = 202/214) stated that some level of improvement in their ability would be meaningful for at least one item, with 5% disagreeing (*n* = 10/214) and 1% unsure (*n* = 2/214). The two most frequently reported reasons for disagreement included “not considering a skill critical for everyday life” (*n* = 5) and “rarely needing to use the movement” (*n* = 3). No open-ended answers were provided by participants who responded “don't know.”

### Part 3. Quantitative Analysis Results Using the Validated MFM32 Assessment

As described in Table 1, all cross-sectional Spearman's rank correlation coefficients between the MFM32 and the target anchors of CGI-C, RULM, SMAIS-ULM and EQ-5D-5L exceeded |*r*| = 0.50, while change from baseline to Week-52 correlations ranged from |*r*| = 0.20–0.50. Evaluation of anchor suitability through empirical CDF plots (11) indicated that the anchor groups adequately discriminated between individuals improving and worsening on the MFM32 (i.e., there was no overlap between improved and worsened groups).

Table 3 shows the LS mean change from baseline (and standard error) to Week 52 on the MFM32 associated with each anchor level. Table 4 details the SEM, 0.5 and 0.2 SD estimates at baseline. These results suggest that overall, ~3 points (3.125%) is a meaningful improvement at the individual patient level in individuals with Type 2 and non-ambulant Type 3 SMA [range: 2.35–3.72 points for anchor-based methods (median age range of 5–8 years old across anchor groups); Table 3].

**Table 3:** LS mean change (standard error) from baseline to Week 52 in MFM32 by anchor group.

**Anchor**	**LS mean change on MFM32 total** **score (% points)**
CGI-C (minimally, much and very much improved groups)	3.49 (0.47) (*n* = 76, mean = 8 years, median = 6 years)
RULM (≥2-points change)	3.11 (0.49) (*n* = 73, mean = 7 years, median = 6 years)
RULM (≥3-points change)	3.72 (0.56) (*n* = 53, mean = 7 years, median = 5 years)
SMAIS-ULM CG (≥3-points change)	2.35 (0.58) (*n* = 51, mean = 9 years, median = 8 years)
EQ-5D-5L CG self-care item (improved by 1 category)	2.88 (0.66) (*n* = 41, mean = 8 years, median = 6 years)

*LS, Least Squares; MFM32, 32-item Motor Function Measure; CGI-C, Clinical Global Impression of Change Scale; RULM, Revised Upper Limb Module; SMAIS-ULM CG, SMA Independence Scale Upper Limb Module Caregiver Report; EQ-5D-5L CG, EuroQol 5D-5L Caregiver Report*.

**Table 4:** Distribution-based MFM32 baseline results.

**Distribution-based method**	**2–25 years MFM32 total** **score (% points)**
±1 SEM	3.26 (*n* = 174)
0.5 SD	5.73 (*n* = 180)
0.2 SD	2.29 (*n* = 180)

*MFM32, 32-item Motor Function Measure; SEM, Standard Error of Measurement; SD, Standard Deviation*.

## Discussion

This study represents a comprehensive multi-pronged approach to evaluating individual patient-level meaningful change assessed using a patient friendly version of the MFM32 (Parts 1 and 2) and the validated MFM32 (Part 3) in individuals with Type 2 and Type 3 SMA. The combined qualitative and quantitative approaches represent endorsed methods, ensuring inclusion of the patient voice via the collection of interview and survey data using patient-friendly versions of the MFM32 (14, 32) as well as traditional quantitative methods using the validated MFM32 clinical assessment (11).

The individuals with SMA and caregivers enrolled in the qualitative interview and survey sample confirmed that stability on the concepts assessed in the patient-friendly MFM32 over 1 year is a meaningful outcome across SMA types and functional status (i.e., ambulant and non-ambulant individuals). This finding supports the importance of examining ≥0 points change on MFM32 as a meaningful change threshold. This is in line with previous interview and survey findings that have included children, adolescents and adults with SMA and have similarly found that maintaining current ability is a meaningful outcome [e.g., Voices of the Patient Report SMA 2018 (33), Rouault et al. (34), McGraw et al. (13), SMA EU Survey 2020 (35), Wan et al. (36)]. When considering improvement, the qualitative interviews also demonstrated that changes to multiple items were deemed potentially more impactful to daily life than a single larger change on an individual item.

Triangulation of the anchor-based estimates in individuals with Type 2 and non-ambulant Type 3 SMA with a median age range of 5–8 years old across anchor groups, confirmed that ~3 points (3.125% points) is a meaningful improvement at the individual patient level using the validated MFM32 assessment. This could be considered a marked level of change as the anchors selected assessed change scores of a previously identified important magnitude on patient and clinical outcomes. The application of this estimate is intended to apply to responder analyses which seek to demonstrate the proportion of patients who experience a meaningful change over a predetermined time point at the individual patient level (37). While anchors related to “no change” groups could have been utilized given the importance of stabilization in this population, anchors associated with improvement were prioritized in order to establish a marked or more substantial score change on the MFM32 in this population. From a quantitative perspective, there is no existing anchor-based meaningful change estimate on the MFM32 in SMA. However, previous anchor-based estimates of meaningful change on the MFM32 in congenital muscular dystrophy using a self-reported global item found that 2.5 points was a within-patient meaningful improvement (38). This result is broadly consistent with the findings in this research.

While not patient centered in nature, the distribution-based SEM estimate also converged on ~3 points as a meaningful change on the MFM32. More variable estimates from the 0.5 and 0.2 SD methods were observed. Previous distribution-based estimates in SMA using a real-world dataset found 3–4 points as an appropriate threshold (Trundell et al., 2019; poster presented at Cure SMA). Of note, distribution-based estimates have previously formed the basis for estimations of meaningful change for motor function scales in SMA such as the Hammersmith Functional Motor Scale Expanded and RULM (39). However, as described by Vazquez-Costa et al. (40), distribution-based estimates approximate measurement error and as such anchor-based methods are required to understand meaningful change.

The individuals included in the anchor-based analyses had a median age range of 5–8 years old across anchor groups and therefore the estimate of 3 points is most relevant for this younger age group. From a clinical point of view, it has been established that the degree of change that is meaningful may vary based on age, with smaller changes likely to be more impactful to older individuals with a longer disease duration compared with younger patients (41). Available natural history data indicate small improvements of motor function on measures such as the MFM32 in children up to 5 years old are possible and are generally followed by a decline after the age of 6 years old (42). For adolescents and adults who understand the impact of the progressive nature of SMA and its impact on daily life, maintaining current abilities has been described as critical (36). This finding is consistent with the qualitative work generated in this study. In this context, it is clear that patient expectations of meaningful change and the capacity to change differs across age groups and disease duration; therefore, a single meaningful change estimate for children, adolescents and adults is not appropriate and while improvement beyond maturational development is the goal for infants and children, stabilization is often the goal for adolescents and adults.

The anchor-based approaches described here included both clinician and caregiver measures, which is a strength of the research. The clinician-reported outcomes (CGI-C, RULM) measure the clinician's perspective on overall health and motor function ability. The aim of this work was to provide a patient-centered perspective of meaningful change on the MFM32 and as such, the SMAIS-ULM caregiver-reported outcome and EQ-5D-5L caregiver report were also used as anchors. When using these anchors, the meaningful change estimates on the MFM32 were smaller and closer to an improvement of 2–3 points. However, there was a weaker correlation between the MFM32 and these outcomes when compared with the clinician-reported outcomes and thus these anchors represent a less appropriate external criterion, despite their inherent patient relevance.

A limitation of the qualitative and survey parts of the study is that the research was conducted in only European and North American countries. Future research should be conducted in additional geographic areas as meaningful change on the MFM32 may vary by culture and geography. In addition, the MFM32 assessment, which is typically administered and rated by physiotherapists, was self-assessed by participants based on a patient-friendly version of the instrument in the qualitative interview and online survey. Due to the involvement of clinical experts and patient groups in the creation of this lay language, it was possible to ensure consistency with the concepts assessed in the clinically validated version of the MFM32, as shown in Supplementary Table 1. However, it is acknowledged that future research should seek to replicate the qualitative findings reported here. This could be achieved by discussing meaningful change with patients and caregivers in the context of MFM32 scores derived from the validated assessment. A further potential limitation of this work relates to the majority of the interview sample including individuals who received treatment or caregivers of treated individuals, while the online survey sample had an even distribution of treated and treatment-naïve patients, or caregivers of individuals. Although individuals were deemed to have had sufficient time on treatment to understand the new trajectory of disease progression, the perspective of meaningful change could have been influenced when compared with treatment-naïve individuals. However, given the results of the online survey, which did not indicate a difference between treated and untreated patients, this limitation is deemed to have been largely alleviated.

The main limitation of the quantitative research was that anchor and distribution-based analyses were only performed in a non-ambulant cohort (unable to walk unassisted for 10 meters or more). Moreover, the anchor-based estimates focused on the total sample population aged 2–25 years old because in certain age subgroups <10 patients were included meaning robust conclusions could not be drawn. Minimal clinically meaningful change is derived specifically for similar cohorts. Future studies with a larger sample size should consider assessing meaningful change on the MFM32 by age and additional relevant clinical subgroups such as individuals who are able to walk and those who are not able to sit. In addition, exploring the effect of scoliosis and contractures in non-ambulant patients could provide additional insights.

## Conclusions

Based on insights from individuals with SMA and caregivers, maintaining functional ability based on a patient-friendly version of the MFM32 is a meaningful outcome to patients and their families. When considering improvement, single-point changes across multiple items is important. The quantitative data using the validated MFM32 assessment and using multiple anchors in a sample with a median age range of 5–8 years old across anchor groups, demonstrated that a ~3-point improvement is meaningful in individuals with Type 2 and non-ambulant Type 3 SMA. Overall, the qualitative and quantitative findings from this study support the importance of examining a range of meaningful change thresholds on the MFM32 including ≥0 points change reflecting stabilization or improvement and ≥3 points change reflecting a higher threshold of improvement.

## Data Availability Statement

Data from the qualitative interviews and online survey: Individual patient level data generated from this study are not publicly available; aggregated data may be provided by the authors upon reasonable request. Requests to access the aggregated data should be directed to the corresponding author.

Data used for the anchor and distribution-based analyses from the SUNFISH Part 2 clinical trial: Qualified researchers may request access to individual patient-level data through the clinical study data request platform (https://vivli.org/). Further details on Roche's criteria for eligible studies are available here (https://vivli.org/members/ourmembers/). For further details on Roche's Global Policy on the Sharing of Clinical Information and how to request access to related clinical study documents, see here (https://www.roche.com/research_and_development/who_we_are_how_we_work/clinical_trials/our_commitment_to_data_sharing.htm).

## Ethics Statement

Ethical approval and oversight of the qualitative interview study was provided by Copernicus Group Independent Review Board, a centralized IRB in the US (Approval Reference Number IRB 20192527). Written informed consent was obtained from participants aged ≥18 years old and written informed consent was provided by caregivers for individuals aged 12-17 years old prior to the interviews, as well as obtaining assent from adolescents. The online survey was conducted following market research principles and hence did not receive ethical approval but was consistent with British Healthcare Business Intelligence Association guidelines for Adverse Event Reporting in Market Research as well as General Data Protection Regulation guidelines and guidelines set by ESOMAR and EphrMRA for all European research. A link to the survey was sent to eligible participants where participants completed a tick box indicating consent to participate prior to survey completion. Assent was provided by caregivers prior to survey completion for individuals aged <18 years old. For SUNFISH Part 2, the ethics statement can be found in Mercuri et al. (17), in press. Written informed consent to participate in this study was provided by the participants' legal guardian/next of kin.

## Author Contributions

TD, HS, JB, AB, TW, and CV have contributed to the material preparation, study design and interpretation for the qualitative interviews, online survey, and quantitative analyses. SR and LY have contributed to the qualitative interviews (SR) and online survey (LY) material preparation, study design, data collection, and interpretation. RC and NG have contributed to the qualitative interview and online survey material preparation, study design, and interpretation. BT, LG, and KG have contributed to the design, analysis and interpretation of the quantitative results. All authors contributed to the development of the first draft of the manuscript and have read and approved the final manuscript.

## Funding

The qualitative interviews, online survey and the exploratory analyses conducted using SUNFISH Part 2 clinical trial data (NCT02908685) described in this manuscript were funded by F. Hoffmann-La Roche. Funding was provided by Roche Products Ltd. and F. Hoffmann-La Roche in the form of salaries for Roche employees, and consultancy fees paid to Adelphi Values (qualitative interviews) and Charles River Associates Inc. (survey).

## Conflict of Interest

HS, JB, and TW are employees and shareholders of Roche Products Ltd. KG is an employee and shareholder of F. Hoffmann-La Roche Ltd. BT is an employee and shareholder of Genentech. TD serves on advisory boards and receives consultancy fees for Roche, Genentech, Biogen, Novartis and Cure SMA. CV is a PI for Roche clinical trials and has received consultancy fees from Roche, Biogen, and Avexis. TD, CV, and AB received consultancy fees from Roche for this project. NG is a volunteer of SMA Europe and SMA Schweiz, mother of a child living with SMA advisor and lecturer for Novartis Gene Therapies (AveXis) Biogen, Novartis, and Roche. RC was an employee with Cure SMA at the time of the study. LG is an employee of Analystat Corporation working in US Medical Affairs at Genentech and Roche as a statistical consultant and has received payment from Genentech Inc. SR is an employee of Adelphi Values, a health outcomes research agency, commissioned and paid by Roche to conduct the qualitative interview part of the study. LY is an employee of Charles River Associates, commissioned and paid by Roche to conduct the online survey part of the study. The authors declare that this study received funding from Roche Products Ltd/F. Hoffmann-La Roche. The funder (F. Hoffmann-La Roche) had the following involvement: qualitative interviews and survey parts of the study, design of the study and interpretation of the data. The funder (F. Hoffmann-La Roche) had the following involvement in the SUNFISH part 2 post-hoc analyses: design, analysis and interpretation of the data. Medical writing and editorial support were funded by F Hoffmann-La Roche.

## Publisher's Note

All claims expressed in this article are solely those of the authors and do not necessarily represent those of their affiliated organizations, or those of the publisher, the editors and the reviewers. Any product that may be evaluated in this article, or claim that may be made by its manufacturer, is not guaranteed or endorsed by the publisher.
