# Supplementary material for: A Patient-Centered Evaluation of Meaningful Change on the 32-Item Motor Function Measure in Spinal Muscular Atrophy Using Qualitative and Quantitative Data
Source: Front Neurol. 2022 Jan 17;12:770423. doi: 10.3389/fneur.2021.770423 (PMC8802297; doi:10.3389/fneur.2021.770423)
Supplement: Supplementary file 1 [file Table_1.docx]

**Supplementary Materials**

**Supplemental Methods:** Adapted from Duong T. et al. BMC Neurol. (2021) 21:143. doi: 10.1186/s12883-021-02166-z.

**Eligibility criteria for the qualitative interviews and online survey**

Participants were identified with support from patient advocacy groups and via Rare Patient Voice. Notable eligibility criteria included the individual with SMA being treatment naïve or in the maintenance dosing phase of nusinersen (SPINRAZA^®^) treatment. Exclusion criteria included being enrolled in a clinical trial or receiving risdiplam (EVRYSDI^®^) or onasemnogene abeparvovec (ZOLGENSMA^®^).

Participants in the maintenance dosing phase of nusinersen treatment were considered eligible since their treatment duration was substantial. These individuals were considered to be treated for a sufficient duration to understand their current functional ability and that this would not influence their perspective of meaningful change. In contrast, patients who had received onasemnogene abeparvovec were excluded as its approval was too close in proximity to the start of this study. Therefore, the patients’ perception of functional ability was deemed to be potentially changing which may have affected their perspective of meaningful change.

**Supplementary Table 1.** Patient friendly MFM32 items and clinical MFM32 items

| **MFM32 item number** | **Clinician-reported MFM32 item**  *(maximum score based on MFM32 User Manual 2^nd^ Edition (2009)* | **MFM32 patient-friendly item presented in the online survey and the qualitative interviews, respectively**  *N.B. Where differences exist between the interview and online survey items, both items are presented.* |
| --- | --- | --- |
| **1** | Supine, hold head for 5 seconds in midline and turns completely from one side to another (D2) | When lying on your back, can you hold your head for 5 seconds and turn it from side to side? |
| **2** | Supine, raises the head and maintains for 5 seconds (D2) | When lying on your back, can you lift your head and keep it lifted for 5 seconds? |
| **3** | Supine, flexes hip and knee more than 90 degrees by raising foot (D2) | When lying on your back, can you bring one knee to your chest? |
| **4** | Supine, leg supported, plantar flexion to dorsiflexion of foot to 90 degrees (D3) | When lying on your back, can you go from pointing your toes to flexing your foot? |
| **5** | Supine, raises hand and moves to the opposite shoulder (D2) | When lying on your back, can you bring one hand to the opposite shoulder? |
| **6** | Supine, legs half flexed, raises pelvis (D1) | When lying on your back, with your knees bent and your feet on the floor slightly apart, can you lift your hips up from the floor and hold for 5 seconds?  When lying on your back, with your knees bent and your feet on the floor slightly apart, can you lift your pelvis up from the floor and hold for 5 seconds? |
| **7** | Supine to prone and frees upper limb from trunk (D2) | When lying on your back, can you turn over onto your stomach and free both of your arms? |
| **8** | Supine, sits up without upper limb support (D1) | When lying on your back, can you sit up without any extra help/support? |
| **9** | Seated on mat, maintains seated position for 5 seconds unsupported (D2) | When you are sitting, can you maintain a seated position and keep contact between the palms of your hands, without any extra help/support?  When seated on the floor, can you maintain a seated position and keep contact between your hands, without any extra help/support? |
| **10** | Seated on mat, leans forward to touch tennis ball (D2) | When you are sitting, can you lean forward to touch an object, without any extra help/support?  When you are sat down, can you lean forward to touch a tennis ball, without any extra help/support? |
| **11** | Seated on mat, stands up without upper limb support (D1) | Can you stand up from sitting on the floor without any extra help/support? |
| **12** | Standing to sitting on chair without upper limb support (D1) | Can you sit down on a chair from standing without any extra help/support? |
| **13** | Seated on chair with no upper limb support for 5 seconds (D2) | Can you sit on a chair for 5 seconds with your head/body in the center? |
| **14** | Seated, from head in flexion, raises and maintains for 5 seconds in midline (D2) | When seated and looking at the floor, can you lift your head up and keep it lifted for 5 seconds?  When seated on the floor, starting from looking at the floor, can you lift your head up and keep it lifted for 5 seconds? |
| **15** | Seated, forearms but not elbows on table, place both hands on top of head, with trunk and head remaining in midline (D2) | When you are sitting down, with your forearms but not elbows on the table, can you bring your arms up to put both hands on top of your head, without moving your body?  When you are sat down, with your forearms but not elbows on the table, can you bring your arms up to put both hands on top of your head, without moving your body? |
| **16** | Seated on chair, without moving the trunk, reaches the pencil with one hand and forearms/hand off the table and elbow in full extension (D2) | When you are sitting down with your forearm on the table, can you touch a pencil in front of you, without moving your body?  When you are sat down with your forearm on the table, can you touch a pencil in front of you, without moving your body? |
| **17** | Pick up 10 coins in one hand (D3) | When you are sitting down with your forearm on the table, can you pick up 10 coins in your hand and hold them?  When you are sat down with your forearm on the table, can you pick up 10 coins in your hand in 20 seconds and hold them? |
| **18** | Seated, traces edge of CD without hand support on table (D3) | When you are sitting down with your forearm on the table, can you trace the edges of a circle using your finger?  When you are sat down with your forearm on the table, can you trace the edges of a CD? |
| **19** | Pick up pencil and draw continuous series of loops inside the frame (D3) | When you are sitting down with your elbow on or off the table, can you pick up a pencil in front of you and draw loops inside a box?  When you are sat down with your elbow on or off the table, can you pick up a pencil and draw loops inside a frame? |
| **20** | Tear sheet of paper folder in 4 (D3) | Can you tear a sheet of paper that has been folded in half and then in half again? |
| **21** | Seated, picks ball up and turns hand over completely (D3) | When you are sitting down with your forearm on the table in front of you, can you pick up a ball directly in front of you and turn your hand over?  When you are sat down with your forearm on the table, can you pick up a ball in front of you and turn your hand over? |
| **22** | Seated, raises finger and touches 8 drawings successively without touching the lines (D3) | When you are sitting down, can you lift your finger and trace the border of a postcard in front of you?  When you are sat down, can you lift your finger and touch the drawings in front of you without touching the lines? |
| **23** | Seated, places two forearms and/or the hands on the table at the same time without moving trunk (D2) | When you are sitting down, starting with your hands by your sides, can you lift your arms to place both forearms/hands on the table at the same time while keeping your body still/stable?  When you are sat down, starting with your hands by your sides, can you lift your arms to place both forearms/hands on the table at the same time while keeping your body still? |
| **24** | Seated on chair, stands up without upper limb support (D1) | Starting seated on a chair, can you stand up without using your arms for support? |
| **25** | Standing without upper limb support for 5 seconds (D1) | Can you stand up without using your arms for support for 5 seconds? |
| **26** | Standing without upper limb support, raises the foot for 10 seconds (D1) | When standing up, can you lift your foot from the floor for 10 seconds without support? |
| **27** | Standing, without support bends to touch the floor with one hand and stands up again (D1) | When standing, can you bend down to touch the floor and stand up again without any help/support? |
| **28** | Standing without support, takes 10 steps forward on both heels (D1) | Can you stand and take 10 steps forward on both heels? |
| **29** | Takes 10 steps forward on a line without support (D1) | Can you take 10 steps forward on in a straight line without support? |
| **30** | Standing, runs 10 meters (D1) | Can you run 10 meters? |
| **31** | Standing on one foot without support, hops 10 times (D1) | Can you hop on one leg 10 times without help/support? |
| **32** | Squat without upper limb support twice in a row (D1) | Can you squat (crouch or sit with knees bent) and then stand back up again twice in a row? |

N.B. This table was adapted from Duong T. et al. BMC Neurol. (2021) 21:143. doi: 10.1186/s12883-021-02166-z. The items in the patient-friendly version of the MFM32 was also reordered based on prior Rasch measurement theory analyses by increasing level of difficulty.
